# Feelings of loneliness, COVID-19-specific-health anxiety and depressive symptoms during the first COVID-19 wave in Swiss persons with multiple sclerosis

**DOI:** 10.1038/s41598-022-22445-0

**Published:** 2022-10-24

**Authors:** Robert Hoepner, Stephanie Rodgers, Katharina Stegmayer, Nina Steinemann, Christina Haag, Pasquale Calabrese, Zina-Mary Manjaly, Anke Salmen, Jürg Kesselring, Chiara Zecca, Claudio Gobbi, Milo A. Puhan, Sebastian Walther, Viktor von Wyl

**Affiliations:** 1 Department of Neurology, Inselspital, Bern University Hospital and University of Bern, Bern, Switzerland; 2 Epidemiology, Biostatistics and Prevention Institute, University of Zurich (UZH), Zürich, Switzerland; 3 Translational Research Center, University Hospital of Psychiatry, University of Bern, Bern, Switzerland; 4 Institute for Implementation Science in Health Care, University of Zurich (UZH), Zurich, Switzerland; 5 Division of Molecular and Cognitive Neuroscience, University of Basel, Basel, Switzerland; 6 Department of Neurology, Schulthess Clinic, Zürich, Switzerland; 7 Department of Health Sciences and Technology, Eidgenössische Technische Hochschule (ETH) Zurich, Zürich, Switzerland; 8 Department of Neurology and Neurorehabilitation, Rehabilitation Centre Kliniken Valens, Valens, Switzerland; 9 Multiple Sclerosis Center, Neurocenter of Southern Switzerland, EOC, Lugano, Switzerland; 10 Faculty of Biomedical Sciences, Università Della Svizzera Italiana (USI), Lugano, Switzerland

**Keywords:** Psychology, Neurology, Risk factors

## Abstract

The aim of our study was to investigate whether self-reported feeling of loneliness (FoL) and COVID-19-specific health anxiety were associated with the presence of depressive symptoms during the first coronavirus disease 2019 (COVID-19) wave. Questionnaires of 603 persons of the Swiss Multiple Sclerosis Registry (SMSR) were cross-sectionally analyzed using descriptive and multivariable regression methods. The survey response rate was 63.9%. Depressive symptoms were assessed by the Beck Depression Inventory-Fast Screen (BDI-FS). COVID-19-specific health anxiety and FoL were measured using two 5-item Likert scaled pertinent questions. High scoring FoL (2.52, 95% confidence interval (CI) (2.06—2.98)) and/or COVID-19 specific health anxiety (1.36, 95% CI (0.87–1.85)) were significantly associated with depressive symptoms. Further stratification analysis showed that the impact of FoL on depressive symptoms affected all age groups. However, it was more pronounced in younger PwMS, whereas an impact of COVID-19 specific health anxiety on depressive symptoms was particularly observed in middle-aged PwMS. FoL and COVID-19-specific health anxiety were age-dependently associated with depressive symptoms during the first COVID-19 wave in Switzerland. Our findings could guide physicians, health authorities, and self-help groups to better accompany PwMS in times of public health crises.

## Introduction

The coronavirus disease 2019 (COVID-19) pandemic caused by the severe acute respiratory syndrome coronavirus-2 (SARS-CoV-2) is an ongoing major global health concern. For the first time in this century, mandatory public health interventions were broadly introduced by governments in many countries, including Switzerland, to control the pandemic during the first COVID-19 wave in spring 2020^1^. These governmental interventions, like limiting social gatherings and travel, wearing facemasks, as well as complete lockdowns including *inter alia* strict stay at home, work from home advice, closure of all shops except grocery stores, closure of universities, schools and nursery schools and restriction of social contacts, had a major disruptive impact on daily life. Concurrently, a public discussion regarding limited health care resources and the need for triage ensued. This increased the uncertainty of getting access to possible limited intensive care unit capacities in populations with chronic diseases affecting the central nervous system, such as in persons with multiple sclerosis (PwMS), as definitions include *inter alia* “Severe and irreversible neurological event or condition” without giving a strict definition thus leaving room for speculations^2^.

Despite the unquestionable need for such restrictions, the impact of these measures on depressive symptoms and mental health, and the association of COVID-19 specific health anxiety and feeling of loneliness (FoL) with depressive symptoms are still unclear. A cross-sectional study prior to the pandemic based on self-reports found a significantly higher FoL in PwMS compared to healthy controls without MS^3^. Further, the degree of loneliness positively correlated with depressive symptoms^3^. In a recent analysis published by the UK MS Register, PwMS were also more likely to feel lonelier compared to healthy controls. FoL occurred more frequently in PwMS with anxiety and depression compared to those PwMS without. However, the levels of anxiety and depression did not change between the period during the COVID-19 outbreak compared to the previous year^4^. Moreover, general health anxiety was more frequently observed in PwMS^5^ and, if present, had a negative impact on “experienced disability” and generalized anxiety^5^. The effect of general health anxiety on health-related quality of life appears to be independent of physical disability in persons with relapsing remitting MS^6^, who were more afraid of relapses^7^. The UK MS Register study implemented the “General Anxiety Disorder-7 (GAD-7) Scale” to assess health anxiety of PwMS during the COVID-19 outbreak^4^, but data specifically targeting health anxiety related to infection with SARS-CoV-2 during the COVID-19 pandemic were and are still lacking.

Based on our registry data, we aimed to assess the association between FoL and COVID-19 specific health anxiety and depressive symptoms during the first COVID-19 wave. We hypothesized that high scores of both FoL and COVID-19 specific health anxiety would be associated with more severe depressive symptoms, and potentially even trigger them.

## Methods

### Sample

#### Study type and response rate

Our study reports on the results of a survey research investigating depressive symptoms of PwMS during the first COVID-19 wave in Switzerland. This wave led to a national lockdown. Our study was nested in the ongoing Swiss Multiple Sclerosis Registry (SMSR)^8,9^. Participants of the SMSR had the opportunity to either fill in a one-time survey or additionally complete regular, semi-annual surveys with varying topics, such as the COVID-19 survey. Invitations for the online survey were sent out on April 8, 2020 and the data collection for the current sample was closed on June 8, 2020. In total, 1759 invitations were sent out via the SMSR platform. Of the active participants of the SMSR (n = 1014), defined as active participation within the last 12 months, a response rate of 63.9% was achieved.

#### Data Source, study population and ethical approval

The SMSR, previously described elsewhere^8,9^, is a Swiss-based, patient-centered, longitudinal observational study initiated and financially supported by the Swiss MS Society (http://www.ClinicalTrials.gov; identifier: NCT02980640) and operated by the University of Zurich. Study recruitment started in June 2016. Persons aged 18 years and older with a diagnosis of clinically isolated syndrome or any type of MS (including relapsing remitting MS, primary progressive MS and secondary progressive MS) who are treated in Switzerland can participate after providing their informed consent and validation of the MS diagnosis by their treating physician. All questionnaires are self-completed either online or as paper–pencil questionnaires. The Ethics Committee of the Canton of Zurich approved the SMSR (No. PB-2016–00894). All assessments and analysis were performed in accordance with relevant guidelines and regulations.

### Measures

#### Assessment of the main outcome variable depressive symptoms during the first COVID-19 wave

Depressive symptoms were assessed using the 7-item Beck Depression Inventory-Fast Screen Index (BDI)-FS^10^. The BDI-FS is a self-reported questionnaire containing the following seven subscales: (I) sadness, (II) pessimism, (III) past failure, (IV) self-dislike, (V) self-criticalness, (VI) suicidal thoughts or wishes, and (VII) loss of interest. Each item can be scored from 0 (absent) to 3 (severe) leading to a final score ranging from 0 to 21. The presence of clinically relevant depressive symptoms was assumed if the BDI-FS sum score was ≥ 4 points, indicative of at least mild depressive symptoms^10^.

#### Assessment of COVID-19 specific health anxiety and feeling of loneliness during the COVID-19 lockdown and patient related variables

Loneliness and COVID-19 specific health anxiety were each evaluated using pertinent statements that were arranged on a 5-items Likert scale (1: none—5: very severe). The wording of the questions on feelings of loneliness and COVID-19 specific health anxiety was: “How lonely do you feel at the moment?” and “How much do you currently suffer from a fear of having a serious disease (such as coronavirus) in addition to MS, without it already having been diagnosed by a physician?”. Loneliness and COVID-19 specific health anxiety were dichotomized using the cut-off ≥ 3 for defining feeling of loneliness or COVID-19 specific health anxiety, respectively. The cut-off of three was used to ensure that all patients with any feeling of loneliness or COVID-19 specific health anxiety were classified as such in our analysis. This should prevent a selection of extreme phenotypes. The variable age (categorized intro three classes: ≤ 35 years; 36—59 years; ≥ 60 years) was used for stratified analyses. Age and sex were considered as confounder variables in multivariable models.

### Statistical analysis

Continuous variables were presented as means (± 95% confidence interval, 95% CI) or medians (25th/75th percentile) according to the scale of measurement. Categorical variables were reported as frequencies. Comparative statistics were performed using the Mann–Whitney test (MWT) for continuous variables or Chi^2^ test for categorical variables. We ran a first multivariable regression model with BDI-FS scores as the dependent variable and sex, age, FoL group, and COVID-19 specific health anxiety as independent variables. A second multivariable regression model was run separately for the age groups ≤ 35 years, 36–59 years, and ≥ 60 years, respectively. Furthermore within the same set of variables we run a multivariable logistic regression model with BDI-FS dichotomized (BDI-FS < 4 vs. ≥ 4 points).

### Ethics approval and consent to participate

The Ethics Committee of the Canton of Zurich approved the SMSR (No. PB-2016-00894).

## Results

### Study population

In total, 847 PwMS responded to the survey. This survey included both PwMS only participating in this single survey and PwMS, who additionally had taken part in the longitudinal surveys. We focused only on those COVID-19 survey responders with any prior survey participation (that is, before the pandemic) in the SMSR in 2019 (n = 648) as the actual situation might have influenced the response rate of the survey. Of those, n = 603 had complete data sets in all variables needed for the main regression analysis. The mean age was 49.22 years with a female preponderance (70.80%, Table 1).

**Table 1:** Characteristics of the sample of persons with MS.

Variable	Mean (95% CI)	Range	Frequency n (%)	Total (n)
Female sex			427 (70.80)	603
Age (years)	49.22 (48.28–50.16)	21–84		603
**Feeling of loneliness**				
1 = not at all			301 (49.92)	603
2			168 (27.86)	603
3			72 (11.94)	603
4			51 (8.46)	603
5 = very much			11 (1.82)	603
≥ 3			134 (22.22)	603
**Health anxiety**				
1 = not at all			297 (49.25)	603
2			193 (32.01)	603
3			69 (11.44)	603
4			33 (5.47)	603
5 = very much			11 (1.82)	603
≥ 3			113 (18.74)	603
**BDI-FS**				
Mean BDI-FS	2.0 (1.79–2.21)	0–17		603
< 4 points			483 (80.10)	603
≥ 4 points			120 (19.90)	603
**Items**				
Sadness	0.23 (0.19–0.26)	0–3		603
Pessimism	0.33 (0.29–0.38)	0–3		603
Past failure	0.28 (0.23–0.33)	0–3		603
Self-dislike	0.22 (0.18–0.27)	0–3		603
Self-criticism	0.34 (0.29–0.39)	0–3		603
Suicidal thoughts/wishes	0.12 (0.09–0.15)	0–3		603
Loss of interest	0.48 (0.43–0.53)	0–3		603

*BDI-FS* Beck Depression Index Fast Screen, *n* number of observations, *95% CI* 95% confidence interval, *COVID-19* coronavirus disease 2019.

### Feeling of loneliness and COVID-19-specific health anxiety

FoL, defined as a score ≥ 3 points, was present in 134/603 (22.20%) of the survey participants (Table 1). Severity of depressive symptoms was significantly higher in persons with FoL (mean (95% CI, n): 4.10 (3.46–4.74, 134) compared to those without FoL: 1.40 (1.24–1.56, 469, MWT p-value < 0.001). At least mild depressive symptoms (BDI-FS cut off: ≥ 4 points) were significantly more frequent in PwMS who felt lonely (59/134 (44.03%)) compared to those who did not (61/469 (13.00%), Chi^2^ p-value < 0.001; data not tabulated).

COVID-19-specific health anxiety, defined as a score ≥ 3 points, was reported by 113/603 (18.70%; Table 1). Severity of depressive symptoms (mean BDI-FS score (95% CI, n) was higher among PwMS with COVID-19 specific health anxiety 3.52 (2.86—4.18, 113) compared to those without anxiety: 1.65 (1.45–1.85, 490, MWT p-value < 0.001). Similarly, at least mild depressive symptoms (BDI-FS cut off: ≥ 4 points) were more frequent in PwMS with COVID-19-specific health anxiety (44/113 (38.94%) compared to those without anxiety 76/414 (18.36%, Chi^2^ p-value < 0.001; data not tabulated).

### Depressive symptoms

During the first COVID-19 wave, 120/603 (19.90%) reported at least mild depressive symptoms with a homogeneous distribution within the seven subscales of the BDI-FS questionnaire (Table 1). BDI-FS scores increased in parallel with the FoL (Pearson correlation coefficient 0.51, p < 0.001, n = 603) and COVID-19-specific health anxiety (Pearson correlation coefficient 0.33, p < 0.001, n = 603; data not tabulated).

### Associations of feeling of loneliness and COVID-19-specific health anxiety with depressive symptoms during the first COVID-19 wave

Multivariable regression analysis with BDI-FS scores as the dependent variable was run within the sample of PwMS (n = 603), showing that FoL (2.52 (2.06—2.98, (p < 0.001)) and COVID-19-specific health anxiety (1.36 (0.87–1.85, (p < 0.001)) were independently associated with higher BDI-FS scores during the first COVID-19 wave. Stratifying the analysis by age groups demonstrated that a significant effect of FoL was present in all age groups but stronger in PwMS with younger age (≤ 35 years), whereas the effect of COVID-19-specific health anxiety remained only significant in PwMS aged 36–59 years (Table 2).

**Table 2:** Multivariable regression analysis stratified by age groups.

Variable	Coefficient	95% CI	p-value	n
**All**				
Feeling of loneliness (≥ 3)	2.52	2.06–2.98	1.30E-24	603
COVID-19-specific health anxiety (≥ 3)	1.36	0.87–1.85	6.31E-8	
Age	0.01	− 0.01–0.03	0.16	
Sex	− 0.25	− 0.67–0.17	0.24	
**Age group ≤ 35 years**				
Feeling of loneliness (≥ 3)	3.13	1.93–4.32	0.000002	83
COVID-19-specific health anxiety (≥ 3)	0.97	− 0.59–2.53	0.22	
Age	0.04	− 0.11–0.21	0.65	
Sex	0.26	− 1.25–1.77	0.73	
**Age group 36–59 years**				
Feeling of loneliness (≥ 3)	2.33	1.76–2.90	1.16E-14	404
COVID-19-specific health anxiety (≥ 3)	1.47	0.88–2.06	0.000001	
Age	0.02	− 0.02–0.06	0.24	
Sex	− 0.50	− 1.03–0.04	0.07	
**Age group ≥ 60 years**				
Feeling of loneliness (≥ 3)	2.60	1.46–3.74	0.000017	116
COVID-19-specific health anxiety (≥ 3)	1.14	− 0.02–2.30	0.06	
Age	0.01	− 0.06–0.08	0.76	
Sex	0.17	− 0.57–0.92	0.64	

Dependent variable: BDI-FS score during COVID-19 pandemic. Sex is coded as follows: 0 = male/1 = female. Level of significance is adjusted following Bonferroni correction p < 0.0125. A multivariable logistic regression analysis with the relevant dependent variable depression, defined as BDI-FS ≥ 4 points, and the above used independent variable was run, confirming the findings of the multivariable linear regression model (supplementary Table 2).

*95% CI* 95% confidence Interval, *n* number of observations, *COVID-19* coronavirus disease 2019.

## Discussion

Our cross-sectional study investigated the associations of self-reported FoL and COVID-19-specific health anxiety with the presence of depressive symptoms during the first COVID-19 wave in a large registry sample of PwMS in Switzerland. We observed a robust association between high FoL scores and depressive symptoms, which was present in all age groups. A higher COVID-19-specific health anxiety in association with depressive symptoms might have an association with age as it was present in the middle aged group (36–59 years of age), which remains present also using 10-steps age categories (supplementary Table 1).

This study on PwMS during the first COVID-19 wave revealed an unfavorable association between the occurrence of FoL and depressive symptoms. Similarly, in a population study based on a nationally representative socioeconomic panel, the risk for FoL was nonlinearly distributed across age with elevated levels among young adults, but also among the oldest (> 86 years) of their sample^11^. This unfavorable effect for the young age group was explained elsewhere by less stable and committed relationships, typically characterizing this life span^12^. The FoL in very high age groups, on the contrary, might be reinforced by a higher occurrence of losing loved ones and difficulties in making new interpersonal contacts due to increased disability^11^. While our study sample of PwMS did not contain participants older than 84 years, we could not deduce viable conclusions pertaining to this age group. However, the explanation of less stable relationships could possibly also apply to our younger age group of PwMS. Indeed, the negative consequences of less stable relationships might have become more serious during the COVID-19 lockdown due to restrictions resulting in reduced personal contacts and group activities, canceled appointments, and working from home, leading to a drastic limitation in interpersonal exchanges. This assumption is further corroborated by recent COVID-19-specific research examining mental health in the general population.

A systematic review found that belonging to a younger age group (≤ 40 years) was a relevant risk factor for chronic / psychiatric illness during the COVID-19 pandemic^12^. This was partly explained by stressful situations related to the COVID-19 outbreak resulting in disproportional psychosocial burden for this age group: job loss, unpredictability, distress due to cancellation of social events, online courses, postponements of exams, detachment from peer groups and family-related care duties, especially applicable to women, were listed as possible examples^13^. However, further COVID-19-specific studies on PwMS confirmed that depressive symptoms were more severe in this group compared to matched controls without MS^14^.

Interestingly, the associations between COVID-19-specific health anxiety and depressive symptoms were only statistically significant in the middle-age group in our study. This might imply differential vulnerabilities depending on specific stages of life. The threat of getting infected with the virus may be of particular significance, especially if there is a danger of losing loved ones and/or suffering professional disadvantages. Interestingly, a previous study on PwMS in the pre COVID-19 era demonstrated that health anxiety was linked to younger age at onset, shorter time since MS diagnosis, and strongly related to social support^15^, which might reflect the diagnosis of a chronic neurological disease. Results cannot be directly compared as the latter study was carried out before the COVID-19 pandemic phase and thus not assessed COVID-19-specific health anxiety as we did. In the general population, younger age was also associated with higher COVID-19 anxiety. Although the reasons for these particular age differences are still to be clarified^16^ our study may add further evidence that the age distribution of loneliness might follow a bimodal distribution, while COVID-19-specific anxiety might represent an inverted U-shape distribution, showing its climax in the middle age.

Our study has several limitations. First, since our study did not consider a history of psychiatric disorders, we cannot exclude that our findings may be influenced by pre-existing vulnerabilities, since it is known that persons with psychiatric disorders are more prone to depression during the COVID-19 pandemic as seen in both PwMS^17^ and the general population^18^. Also, pre-existing generalized anxiety disorder was not assessed, despite the robust evidence of its comorbidity with depression^19,20^. Therefore, the association between COVID-19-specific anxiety and depressive symptoms could also be a result of these co-occurring disorders, and MS or COVID-19 specific statements cannot be drawn. Second, this was a cross-sectional study, limiting causal inferences. In addition, the complex inter-relations between FoL, COVID-19-specific anxiety, and depressive symptoms were also not considered and therefore some interactive effects could not be fully disentangled by our study design. In particular, a circularity of FoL might have been present. However, as FoL has been established as relevant risk factor of depression in pooled data^21^, the overlap does not exclude associations. Further both scales used for evaluation of FoL and COVID-19-specific health anxiety as well as the cut-off of ≥ 3 points on the corresponding Likert-scales have not been validated before, which should be taken into account while interpreting our data. Furthermore, the age stratification has to be highlighted which was mainly based on group sizes and run in two different settings to mutually validate each other. Moreover, in this regards we are adhering to open data policy and are willing to share our data set with others upon reasonable request.

Further, as social support and self-perceived loneliness are interrelated, it is likely that persons lacking social support also experience high levels of FoL^22,23^. The same holds for certain socio-demographic variables, such as education and civil status, which were excluded as they could change over time and were not available for all PwMS at the time of the first COVID-19 wave. Finally, we could neither determine whether the results differed from the year before the pandemic outbreak nor whether they were COVID-19- or MS-specific, as this appears to be the case in the general population. A healthy control group would be required to answer this latter question, which is not within the scope of the SMSR.

## Conclusions

Our findings on PwMS indicate that FoL and COVID-19-specific health anxiety might be age-dependent factors associated with depressive symptoms during the first COVID-19 wave in Switzerland. Even though our cross-sectional data do not allow causal conclusions for the development of depression, it seems plausible that FoL and COVID-19-specific health anxiety might be associated with depressive symptoms and might represent targets for interventions. Hence, these differential vulnerabilities depending on specific periods of life require particular consideration regarding the needs of these subgroups of PwMS. As a consequence, while the focus of support for younger PwMS should be on maintaining social contacts, more emphasis must be placed on information about COVID-19-related health issues, to prevent affective disturbances emerging from lockdown-constraints in older subjects.

Additional studies focusing on age-dependent needs of PwMS are required to better prevent and treat depression in PwMS. These findings could be of specific interest for physicians, health authorities, and self-help groups to advance the support of PwMS during public health crises.

## Supplementary Information

Supplementary Tables.

## Data Availability

The datasets used and/or analysed during the current study are available from the corresponding author on reasonable request.
