# Supplementary material for: Feelings of loneliness, COVID-19-specific-health anxiety and depressive symptoms during the first COVID-19 wave in Swiss persons with multiple sclerosis
Source: Sci Rep. 2022 Oct 24;12:17829. doi: 10.1038/s41598-022-22445-0 (PMC9591317; doi:10.1038/s41598-022-22445-0)
Supplement: Supplementary file 1 — Supplementary Tables. [file 41598_2022_22445_MOESM1_ESM.docx]

**Supplementary Info**

| **Variable** | **Coefficient** | **95%CI** | **p-value** | **n** |
| --- | --- | --- | --- | --- |
| **Age group 21-30 years** |  |  |  |  |
| Feeling of loneliness (≥ 3) | 3.02 | 1.13-4.91 | **0.003** | 33 |
| COVID-19-specific health anxiety (≥ 3) | 0.14 | -2.41-2.68 | 0.914 |  |
| Age | -0.02 | -0.39-0.35 | 0.911 |  |
| Sex | -0.50 | -2.58-1.57 | 0.623 |  |
| **Age group 31 – 40 years** |  |  |  |  |
| Feeling of loneliness (≥ 3) | 2.19 | 1.08-3.30 | **0.00016** | 121 |
| COVID-19-specific health anxiety (≥ 3) | 1.57 | 0.31-2.82 | 0.02 |  |
| Age | 0.06 | -0.14-0.25 | 0.57 |  |
| Sex | -0.07 | -1.35-1.22 | 0.92 |  |
| **Age group 41 -50 years** |  |  |  |  |
| Feeling of loneliness (≥ 3) | 1.91 | 1.15-2.67 | **1.64673560829708E-06** | 168 |
| COVID-19-specific health anxiety (≥ 3) | 0.94 | 0.16-1.72 | 0.02 |  |
| Age | 0.09 | -0.02-0.19 | 0.10 |  |
| Sex | -0.18 | -0.84-0.47 | 0.58 |  |
| **Age group 51 -60 years** |  |  |  |  |
| Feeling of loneliness (≥ 3) | 2.86 | 1.94-3.78 | **5.54976841516077E-09** | 177 |
| COVID-19-specific health anxiety (≥ 3) | 1.45 | 0.51-2.39 | **0.003** |  |
| Age | 0.05 | -0.09-0.19 | 0.47 |  |
| Sex | -0.70 | -1.55-0.16 | 0.11 |  |
| **Age group 61 -70 years** |  |  |  |  |
| Feeling of loneliness (≥ 3) | 2.38 | 0.96-3.80 | **0.001** | 87 |
| COVID-19-specific health anxiety (≥ 3) | 1.51 | 0.20-2.82 | 0.03 |  |
| Age | -0.03 | -0.18-0.11 | 0.65 |  |
| Sex | 0.04 | -0.82-0.90 | 0.93 |  |
| **Age group 71 -80 years** |  |  |  |  |
| Feeling of loneliness (≥ 3) | 4.38 | 2.30-6.46 | **0.001** | 15 |
| COVID-19-specific health anxiety (≥ 3) | n.a. |  |  |  |
| Age | 0.04 | -0.15-0.24 | 0.65 |  |
| Sex | 1.27 | 0.29-2.25 | 0.02 |  |
| **Age group 81 -90 years** |  |  |  |  |
| Feeling of loneliness (≥ 3) | L.c. |  |  | 2 |
| COVID-19-specific health anxiety (≥ 3) | L.c. |  |  |  |
| Age | L.c. |  |  |  |
| Sex | L.c. |  |  |  |

Supplement table 1: Multivariable regression analysis stratified by 10-steps age categories. Dependent variable: BDI-FS score during COVID-19 pandemic. Sex is coded as follows: 0=male/1=female. Abbreviations: 95%CI: 95% Confidence Interval, n: number of observations, COVID-19: coronavirus disease 2019. Level of significance following Bonferroni correction is defined as 0.007; significant p-values are highlighted in bold. Comment: n.a. means not possible to calculate as all values of the variable are the same here COVID-19-specific health anxiety <3 points. Lc. means no regression analysis run due to low number of cases.

| **Variable** | **Exp(B)** | **95%CI** | **p-value** | **n** |
| --- | --- | --- | --- | --- |
| All |  |  |  |  |
| Feeling of loneliness (≥ 3) | 4.66 | 2.96-7.34 | 3.1386E-11 | 603 |
| COVID-19-specific health anxiety (≥ 3) | 2.72 | 1.68-4.40 | 0.000048 |  |
| Age | 1.00 | 0.98-1.02 | 0.87 |  |
| Sex | 0.89 | 0.55-1.44 | 0.64 |  |
| **Age group ≤ 35 years** |  |  |  |  |
| Feeling of loneliness (≥ 3) | 10.85 | 3.03-38.78 | 0.000245 | 83 |
| COVID-19-specific health anxiety (≥ 3) | 1.82 | 0.30-11.19 | 0.52 |  |
| Age | 1.01 | 0.83-1.23 | 0.89 |  |
| Sex | 306775506.5 | 0.00 | 0.998 |  |
| **Age group 36 – 59 years** |  |  |  |  |
| Feeling of loneliness (≥ 3) | 3.74 | 2.18-6.42 | 0.000002 | 404 |
| COVID-19-specific health anxiety (≥ 3) | 2.87 | 1.65-5.0 | 0.000186 |  |
| Age | 1.01 | 0.97-1.05 | 0.59 |  |
| Sex | 0.72 | 0.41-1.26 | 0.25 |  |
| **Age group ≥ 60 years** |  |  |  |  |
| Feeling of loneliness (≥ 3) | 4.76 | 1.28-17.64 | 0.02 | 116 |
| COVID-19-specific health anxiety (≥ 3) | 3.25 | 0.85-12.45 | 0.09 |  |
| Age | 1.01 | 0.91-1.13 | 0.82 |  |
| Sex | 1.05 | 0.34-3.21 | 0.94 |  |

Supplement table 2: Multivariable logistic regression analysis stratified by age groups. Dependent variable: BDI-FS score (≥4 vs. <4 points) during the COVID-19 pandemic. Sex is coded as follows: 0=male/1=female. Level of significance is adjusted following Bonferroni correction p<0.0125. Abbreviations: 95%CI: 95% Confidence Interval, n: number of observations, COVID-19: coronavirus disease 2019.
